# Supplementary figures and images for: Post-mating parental behavior trajectories differ across four species of deer mice
Source: PLoS One. 2022 Oct 17;17(10):e0276052. doi: 10.1371/journal.pone.0276052 (PMC9576063; doi:10.1371/journal.pone.0276052)

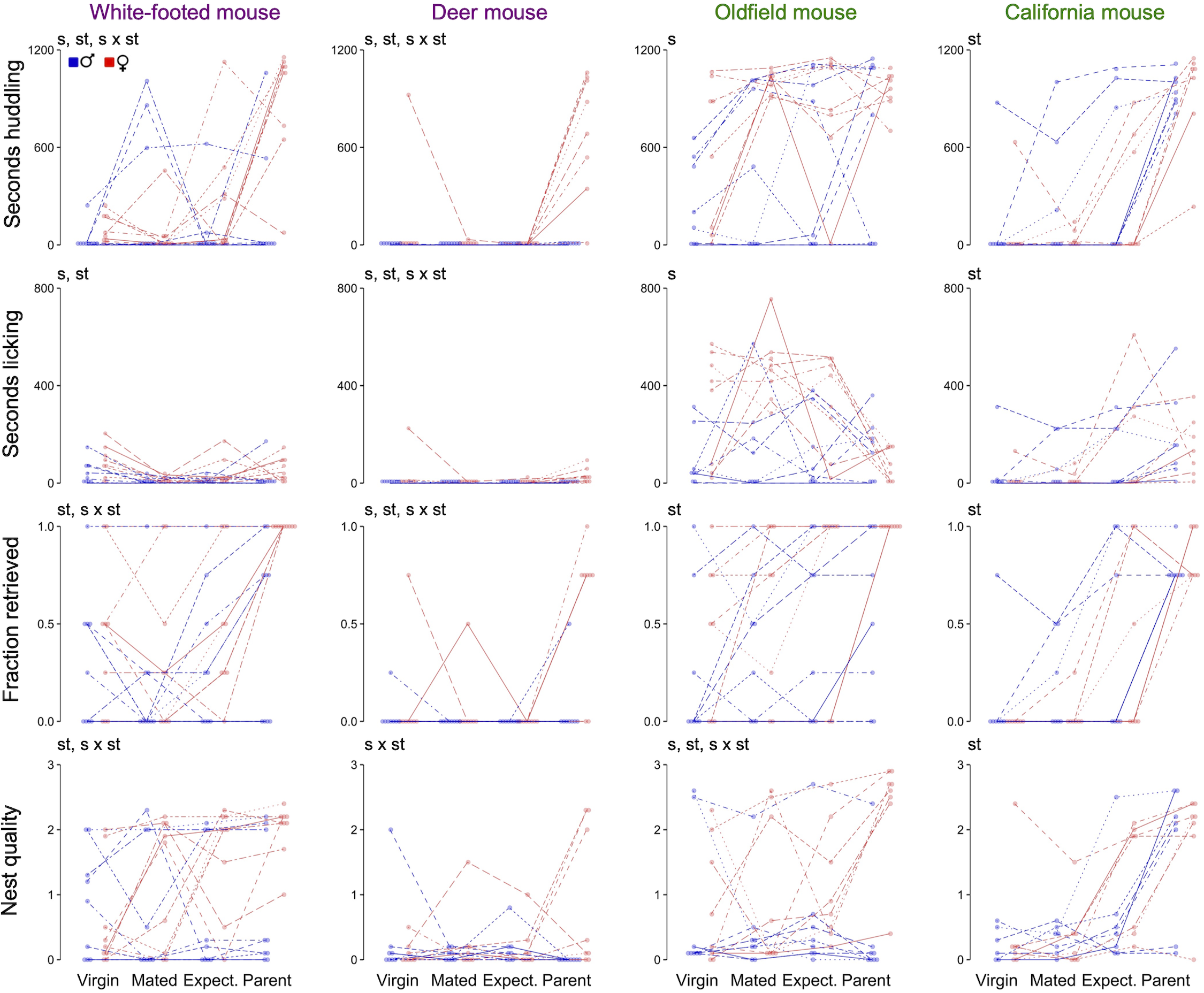

Supplement: S1 Fig — Male (blue) and female (red) trajectories as measured by (A) Time spent huddling (seconds); (B) time spent licking pups (seconds); (C) fraction of pups retrieved to the nest; (D) nest quality score (from 0 to 3). Individuals have unique line types. st, main effect of reproductive state; s, main effect of sex; st × s, interaction between reproductive state and sex by linear mixed models (P < 0.05; see Fig 2 and Materials and methods for additional details). Sample sizes (in pairs): white-footed mouse, n = 9; deer mouse, n = 8; oldfield mouse, n = 8; California mouse, n = 7. (TIFF) [file pone.0276052.s003.tiff]
